# Post-Discharge Risk of Mortality in Children under 5 Years of Age in Western Kenya: A Retrospective Cohort Study

**DOI:** 10.4269/ajtmh.23-0186

**Published:** 2023-08-07

**Authors:** Titus K. Kwambai, Simon Kariuki, Menno R. Smit, Sarah Nevitt, Eric Onyango, Martina Oneko, Sammy Khagayi, Aaron M. Samuels, Mary J. Hamel, Kayla Laserson, Meghna Desai, Feiko O. ter Kuile

**Affiliations:** ^1^ Centre for Global Health Research, Kenya Medical Research Institute, Kisumu, Kenya;; ^2^ Department of Clinical Sciences, Liverpool School of Tropical Medicine, Liverpool, United Kingdom;; ^3^ Division of Parasitic Diseases and Malaria, Center for Global Health, Centers for Disease Control and Prevention, Kisumu, Kenya;; ^4^ Division of Parasitic Diseases and Malaria, Center for Global Health, Centers for Disease Control and Prevention, Atlanta, Georgia;; ^5^ Amsterdam Centre for Global Child Health, Emma Children’s Hospital, Amsterdam University Medical Centres, Amsterdam, The Netherlands;; ^6^ Department of Health Data Science, University of Liverpool, Liverpool, United Kingdom

## Abstract

Limited evidence suggests that children in sub-Saharan Africa hospitalized with all-cause severe anemia or severe acute malnutrition (SAM) are at high risk of dying in the first few months after discharge. We aimed to compare the risks of post-discharge mortality by health condition among hospitalized children in an area with high malaria transmission in western Kenya. We conducted a retrospective cohort study among recently discharged children aged < 5 years using mortality data from a health and demographic surveillance system that included household and pediatric in-hospital surveillance. Cox regression was used to compare post-discharge mortality. Between 2008 and 2013, overall in-hospital mortality was 2.8% (101/3,639). The mortality by 6 months after discharge (primary outcome) was 6.2% (159/2,556) and was highest in children with SAM (21.6%), followed by severe anemia (15.5%), severe pneumonia (5.6%), “other conditions” (5.6%), and severe malaria (0.7%). Overall, the 6-month post-discharge mortality in children hospitalized with SAM (hazard ratio [HR] = 3.95, 2.60–6.00, *P* < 0.001) or severe anemia (HR = 2.55, 1.74–3.71, *P* < 0.001) was significantly higher than that in children without these conditions. Severe malaria was associated with lower 6-month post-discharge mortality than children without severe malaria (HR = 0.33, 0.21–0.53, *P* < 0.001). The odds of dying by 6 months after discharge tended to be higher than during the in-hospital period for all children, except for those admitted with severe malaria. The first 6 months after discharge is a high-risk period for mortality among children admitted with severe anemia and SAM in western Kenya. Strategies to address this risk period are urgently needed.

## RESEARCH IN CONTEXT

### Evidence before this study.

Severe anemia is a major cause of morbidity and mortality in malaria-endemic areas in sub-Saharan Africa. It contributes substantially to in-hospital mortality. However, limited evidence suggests that post-discharge mortality in children hospitalized for severe anemia and other health conditions may be similar to or higher than in-hospital mortality. The magnitude and risk factors for post-discharge mortality among this group of children have not been clearly established in Kenya. We searched PubMed, without language restriction, from inception to November 30, 2022. None of the studies compared the risks of post-discharge mortality by health conditions.

### Added value of this study.

This retrospective cohort study adds to the limited body of evidence on the magnitude and risk factors for post-discharge mortality in malaria-endemic sub-Saharan Africa. Our study utilizes data that were systematically collected over a period of 5 years in a health and demographic surveillance system in a high–disease burden area. We show that children admitted with severe anemia or severe acute malnutrition (SAM) are at increased risk of death in the initial 6 months after discharge compared with children with other health conditions.

### Implications of all the available evidence.

These data emphasize the importance of adequate follow-up and management of children with SAM after discharge. They also support the importance of the newly issued post-discharge malaria chemoprevention (PDMC) guidelines published in June 2022 by the WHO, which specifically target children with severe anemia in malaria-endemic areas.

## INTRODUCTION

All-cause severe anemia is a major public health problem in malaria-endemic areas in sub-Saharan Africa with a complex and multifactorial etiology.^1^

Rates of in-hospital mortality due to severe anemia range from 4% to 12% in different epidemiological settings.^2^^–^^4^ However, several studies have shown that the first few months after discharge are also a high-risk period for further mortality, which can be as high as or higher than in-hospital mortality,^5^^–^^7^ particularly in malaria-endemic areas, possibly reflecting the effects of new or recrudescent malaria infections.^7^^,^^8^

The high risk of post-discharge mortality has received little attention relative to the burden, diagnosis, and care of children during their in-hospital stay.^9^ An improved understanding of the main risk groups of post-discharge mortality is needed to help policymakers develop risk-guided management strategies to reduce pediatric mortality. This retrospective cohort study aimed to compare the magnitude of post-discharge mortality among young children admitted with severe anemia or other health conditions in a high–malaria transmission setting in western Kenya.

## MATERIALS AND METHODS

### Data sources and study setting.

We used data from the health and demographic surveillance system (HDSS) run by the Kenya Medical Research Institute (KEMRI) and U.S. CDC in Siaya County, western Kenya, including both household and pediatric in-hospital surveillance in Siaya County Referral Hospital (formerly Siaya District Hospital). Details about the KEMRI/CDC HDSS and data collected have been previously described.^10^ The household surveillance provided population-based demographic information collected by trained field workers through rounds of home visitations every 4 months. The in-hospital surveillance provided data on all pediatric admissions up to 12 years of age. The in-hospital and household data were linked using the HDSS unique personal identification numbers and fingerprinting^10^ to monitor deaths after hospital discharge.

This retrospective cohort analysis included all children aged < 5 years admitted to the pediatric department from 2008 to 2013 residing within the HDSS study area.^10^ Admissions due to surgery, trauma/injury, malignancy, or sickle cell anemia were excluded.

Each hospital record was examined and categorized by health condition based on the diagnosis on discharge or in-hospital death. If these records were missing, the admission diagnosis was used. Children were categorized into the following four primary health conditions: all-cause severe anemia (hemoglobin (Hb) < 5.0 g/dL or requiring a blood transfusion), severe malaria (microscopy- or rapid diagnostic test–confirmed malaria infection and receipt of parenteral treatment with artesunate or quinine), severe pneumonia, and severe acute malnutrition (SAM). The diagnoses for severe pneumonia and SAM were based on the Kenya Ministry of Health guidelines for clinical management during the study period.^11^ Children who did not have these four diagnoses were pooled under “other conditions.”

The primary outcome was all-cause death within 6 months after discharge. Secondary outcomes included in-hospital mortality. The follow-up time for the in-hospital mortality analysis was defined as the number of days between the date of admission and either the date of in-hospital death or the date of discharge (censoring date) +1. For analysis of post-discharge mortality, this was defined as the number of days between the date after the date of discharge and either the date of death or the date of censoring. Children were censored if they left the study area because of out-migration or reached 5 years of age or if the post-discharge surveillance ended before the event had occurred.

Categorical variables were described as proportions (percentages), and continuous variables were described as mean and corresponding SDs or as median with corresponding interquartile ranges (IQRs). Univariate and multivariable Cox regression analyses were used to analyze time-to-event outcomes with hazard ratios (HRs), and 95% CI were reported for comparisons between groups. Risk factors associated with mortality in the univariate analysis (*P* < 0.2) were included in the multivariable models as covariates. Predictor variables explored included sex, age at admission, bed net use, antibiotic use, blood transfusion, mother’s educational status, mother’s HIV status, socioeconomic status (SES), Hb levels on admission, discharge season, and distance from the hospital. There were insufficient data for the child’s HIV status (available for 5.5% only) to allow its inclusion in any analyses. Two-sided *P* values < 0.05 were regarded as statistically significant.

The Mantel-Haenszel odds ratio (MHOR) for paired binary outcomes was used to compare in-hospital and post-discharge mortality rates (MRs).^12^ Forest plots were used for the graphical presentation of HRs and Kaplan-Meier plots for visualization of the time-to-event outcomes. Data were analyzed using STATA version 14 (College Station, TX).

The household and hospital surveillance of the HDSS was approved by the ethical review boards of the KEMRI (SSC #647) and CDC (IRB #3308). Informed written consent was obtained from compound heads for the participation of their families in all aspects of the HDSS.^10^

## RESULTS

### Characteristics of the study groups.

Between January 1, 2008 and December 31, 2013, 69,492 children living in the combined HDSS and hospital catchment areas met the inclusion criteria. During this period, there were 3,639 hospital admissions involving 4,472 different diagnoses, including severe anemia (*N* = 651), severe malaria (*N* = 1,033), severe pneumonia (*N* = 996), SAM (*N* = 271), and other conditions (*N* = 1,521). The median (IQR) for post-discharge follow-up time was 23.6 (10.3–36.0) months, and the mean (SD) age on admission was 17.8 (13.7) months. Over half (63.3%) of children were reported to have slept under a bed net the night before admission. The baseline characteristics differed between primary health conditions, including the median age at admission and the median duration of follow-up (Table 1). Overall, 136/2,730 (5.0%), 307/2,608 (11.8%), and 595/2,652 (22.4%) of children were lost to follow-up by 3, 6, and 12 months after discharge, respectively (Table 2). To minimize biases related to censoring of participants with missing data due to loss to follow-up,^13^ the maximum duration of follow-up was truncated at 12 months for analysis.

**Table 1:** Baseline characteristics

Characteristic	Severe anemia (*N* = 651)	Severe malaria (*N* = 1,033)	Pneumonia (*N* = 996)	Malnutrition (*N* = 271)	Other conditions (*N* = 1,521)	Total children*
Sex (male), no./total no. (%)	344/651 (52.8)	530/1,033 (51.3)	523/996 (52.5)	139/271 (51.3)	828/1,521 (54.4)	1,926/3,639 (52.9)
First admission age (months), mean ± SD	18.7 ± 13.6	18.6 ± 14.0	12.7 ± 10.9	15.5 ± 9.8	18.7 ± 14.3	17.8 ± 13.7
Bed net use, no./total no. (%)	388/651 (59.6)	884/1033 (85.6)	691/996 (69.4)	189/271 (69.7)	677/1,521 (44.5)	2,829/4,472 (63.3)
Distance (km), median (IQR)	7.4 (4.1–10.3)	6.5 (3.1–9.9)	7.3 (3.5–10.0)	7.7 (4.1–10.7)	6.4 (2.4–10.1)	6.8 (2.9–10.0)
SES, median (IQR)	−0.2 (−0.6 to 0.2)	−0.1 (−0.4 to 0.3)	−0.1 (−0.5 to 0.3)	−0.3 (−0.6 to 0.0)	−0.1 (−0.4 to 0.4)	−0.1 (−0.4 to 0.3)
Mother’s survival status, no./total no. (%)						
Alive	629/651 (96.6)	1,007/1,033 (97.5)	979/996 (98.3)	265/271 (97.8)	1,465/1,521 (96.3)	3,527/3,639 (96.9)
Dead	2/651 (0.3)	4/1,033 (0.4)	6/996 (0.6)	1/271 (0.4)	13/1,521 (0.9)	23/3,639 (0.6)
Unknown	20/651 (3.1)	22/1,033 (2.1)	11/996 (1.1)	5/271 (1.8)	43/1,521 (2.8)	89/3,639 (2.4)
Father’s survival status, no./total no. (%)						
Alive	546/651 (83.9)	865/1,033 (83.7)	860/996 (86.3)	231/271 (85.2)	1,278/1,521 (84.0)	3,092/3,639 (850.)
Dead	28/651 (4.3)	43/1,033 (4.2)	43/996 (4.3)	12/271 (4.4)	68/1,521 (4.5)	157/3,639 (4.3)
Unknown	77/651 (11.8)	125/1,033 (12.1)	93/996 (9.3)	28/271 (10.3)	175/1521 (11.5)	390/3,639 (10.7)
Mother’s HIV status, no./total no. (%)						
Positive	79/651 (12.1)	100/1,033 (9.7)	139/996 (14.0)	50/271 (18.5)	181/1,521 (11.9)	442/3,639 (12.1)
Negative	294/651 (45.2)	525/1,033 (50.8)	467/996 (46.9)	98/271 (36.2)	652/1,521 (42.9)	1625/3,639 (44.7)
Unknown	278/651 (42.7)	408/1,033 (39.5)	390/996 (39.2)	123/271 (45.4)	688/1,521 (45.2)	1,572/3,639 (43.2)
Mother’s educational level, no./total no. (%)						
Lower primary	24/651 (3.7)	28/1,033 (2.7)	40/996 (4.0)	16/271 (5.9)	51/1,521 (3.4)	128/3,639 (3.5)
Upper primary	386/651 (59.3)	609/1,033 (59.0)	587/996 (58.9)	162/271 (59.8)	811/1,521 (53.3)	2,048/3,639 (56.3)
Secondary	217/651 (33.3)	350/1,033 (33.9)	329/996 (33.0)	81/271 (29.9)	550/1,521 (36.2)	1,261/3,639 (34.7)
Tertiary	15/651 (2.3)	29/1,033 (2.8)	30/996 (3.0)	8/271 (3.0)	86/1,521 (5.7)	153/3,639 (4.2)

IRQ = interquartile range; Other conditions = undefined conditions in children who did not have severe anemia, severe malaria, severe pneumonia, or severe acute malnutrition; SES = socioeconomic status. Combination conditions comprise children with a combination of more than one condition including severe anemia, severe malaria, severe pneumonia, severe acute malnutrition, or (multiple) “other conditions.”

* The total number of 3,639 individual children had 4,472 different diagnoses because some children had multiple diagnoses.

**Table 2:** Outcomes

Outcomes	Severe anemia (*N* = 651)	Severe malaria (*N* = 1,033)	Pneumonia (*N* = 996)	SAM (*N* = 271)	Other conditions (*N* = 1,521)	Total*
Duration of hospital stay during initial admission (days), median (IQR)	4 (2–5)	4 (2–5)	4 (3–6)	7 (4–10)	3 (2–5)	3 (2–5)
Discharge during malaria season, no./total no. (%)	307/651 (47.2)	490/1,033 (47.4)	497/996 (49.9)	127/271 (46.9)	738/1,521 (48.5)	1,713/3,639 (47.1)
Follow-up (months)	22.2 (6.9–35.6)	23.3 (11.2–34.3)	24.5 (11.8–35.4)	18.1 (4.1–29.5)	24.7 (11.0–38.4)	23.6 (10.3–36.0)
Loss to follow-up, no./total no. (%)*†						
By 3 months	10/215 (4.3)	35/494 (7.6)	18/544 (3.3)	8/98 (7.3)	65/1,379 (4.7)	136/2,730 (5.0)
By 6 months	27/207 (12.7)	61/491 (14.1)	56/541 (10.9)	16/96 (15.7)	147/1,369 (11.4)	307/2,608 (11.8)
By 12 months	47/200 (24.4)	122/483 (32.8)	111/525 (24.2)	25/95 (26.9)	290/1,349 (25.2)	595/2,652 (22.4)
Deaths, no./total no. (%)						
In-hospital*	15/255 (5.9)	5/499 (1)	16/586 (2.7)	12/130 (9.2)	27/1,468 (1.8)	75/2,938 (2.6)
Risk of death from discharge, no./total no. (%)*‡						
By 3 months	25/230 (10.9)	0/459 (0)	26/552 (4.7)	20/110 (18.2)	62/1,376 (4.5)	133/2,727 (4.9)
By 6 months	33/213 (15.5)	3/433 (0.7)	29/514 (5.6)	22/102 (21.6)	72/1,294 (5.6)	159/2,556 (6.2)
By 12 months	40/193 (20.7)	11/372 (3)	45/459 (9.8)	23/93 (24.7)	92/1,151 (8)	211/2,268 (9.3)
Time to post-discharge death (months), median (IQR)	2.1 (0.5–5.2)	4.7 (1.5–7.8)	2.6 (0.9–6.4)	0.7 (0.2–2.1)	1.3 (0.5–4.6)	1.7 (0.5–5.4)
Cumulative in-hospital and 12-month post-discharge deaths*	55/255 (21.6)	16/499 (3.2)	61/586 (10.4)	35/130 (2.7)	119/1,468 (8.1)	286/2,938 (9.7)
HR for in-hospital mortality, HR (95% CI)	2.64 (1.58–4.41) *P <* 0.001	0.38 (0.22–0.67) *P <* 0.001	0.86 (0.52–1.44) *P* = 0.575	1.84 (1.11–3.05) *P* = 0.018	0.69 (0.37–1.30) *P* = 0.253	–
HR for post-discharge mortality versus all other health conditions, HR (95% CI)						
By 3 months	2.49 (1.64–3.79) *P <* 0.001	0.27 (0.16–0.45) *P <* 0.001	0.84 (0.55–1.29) *P* = 0.426	4.08 (2.60–6.40) *P <* 0.001	0.92 (0.55–1.53) *P* = 0.736	–
By 6 months	2·55 (1.74–3.71) *P <* 0.001	0.33 (0.21–0.53) *P <* 0.001	0.94 (0.63–1.41) *P* = 0.782	3.95 (2.60–6.00) *P <* 0.001	0.99 (0.61–1.59) *P* = 0.955	–
By 12 months	2.29 (1.63–3.22) *P <* 0.001	0.42 (0.29–0.60) *P <* 0.001	0.85 (0.60–1.22) *P* = 0.388	2.81 (1.88–4.20) *P <* 0.001	0.82 (0.54–1.26) *P* = 0.372	–

IQR = interquartile range; SAM = severe acute malnutrition. Other conditions comprise children who did not have severe anemia, severe malaria, severe pneumonia, or severe acute malnutrition. Post-discharge mortality risk (follow-up truncated to 12 months).

* Total number of children contributing includes children with multiple conditions; i.e., 3,639 individual children had 4,472 different diagnoses.

† Denominator excludes deaths.

‡ Denominator excludes losses to follow-up.

### In-hospital mortality.

The median (IQR) length of in-hospital stay was 3 (2–5) days. The overall in-hospital crude MR was 2.8% (101 of 3,639 admissions). Children admitted with SAM had the highest in-hospital mortality (MR = 9.2%) and the longest median (IQR) in-hospital stay (7 [4–10] days). Children with severe anemia had the second highest in-hospital mortality (MR = 5.9%), with a median (IQR) in-hospital stay of 4 (2–5) days. The corresponding in-hospital MRs for severe pneumonia, other conditions, and severe malaria were 2.7%, 1.8%, and 1.0%, respectively (Table 2). In-hospital mortality was significantly higher in children admitted with severe anemia compared with those admitted for other reasons (i.e., all other children admitted without severe anemia but with severe pneumonia, severe malaria, SAM, or other conditions) (HR = 2.64, 1.58–4.41, *P* < 0.001). In-hospital mortality was also higher among children admitted with SAM compared with children without SAM (HR = 1.84, 1.11–3.05, *P* = 0.018) but was similar among children with and without severe pneumonia (HR = 0.86, 0.52–1.44, *P* = 0.575) or with and without “other syndromes” (HR = 0.69, 0.37–1.30, *P* = 0.253). By contrast, in-hospital mortality was lower among children with severe malaria versus those without (HR = 0.38, 0.22–0.67, *P* = 0.001) (Table 2).

### Post-discharge mortality.

Cumulative post-discharge mortality among the 3,538 children discharged alive was 4.9%, 6.2%, and 9.3% by 3, 6, and 12 months after discharge, respectively (Table 2). By 6 months, all-cause post-discharge mortality was highest in the SAM group (MR = 21.6%), followed by severe anemia (MR = 15.5%), severe pneumonia and other conditions (MR = 5.6%), and then severe malaria (MR = 0.7%) (Table 2). Post-discharge mortality by 6 months was significantly higher in children with SAM versus without SAM (HR = 3·95, 2.60–6.00, *P* < 0.001). Similarly, severe anemia was associated with higher mortality compared with children admitted for other reasons (HR = 2.55, 1.74–3.71, *P* < 0.001). By contrast, 6-month post-discharge mortality was similar among children with and without severe pneumonia (HR = 0.94, 0.63–1.41, *P* = 0.782) and with and without other conditions (HR = 0.99, 0.61–1.59, *P* = 0.955) and was lower in children admitted with severe malaria versus those without severe malaria (HR = 0.33, 0.21–0.53, *P* < 0.001) (Table 2).

**Figure 1.**
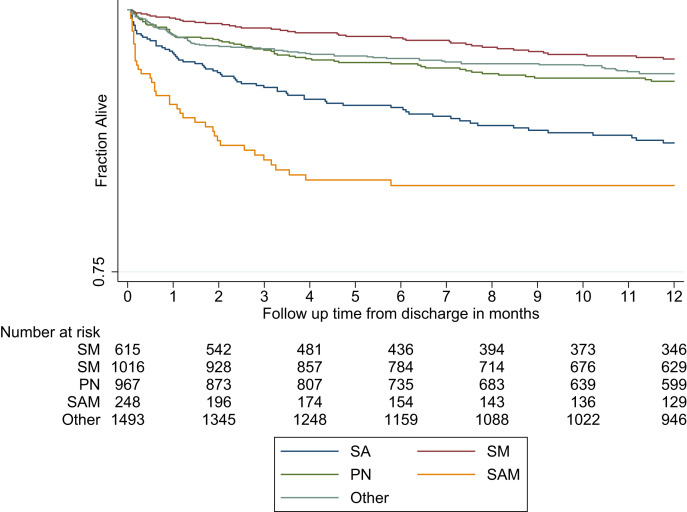
Survival curves showing time to post-discharge death. PN = severe pneumonia; SA = severe anemia; SAM = severe acute malnutrition; SM = severe malaria. “Other” denotes the undefined conditions excluding SA, SM, PN, or SAM.

The Kaplan-Meier plot (Figure 1) illustrates that among the post-discharge deaths by 12 months (*N* = 211/2,268; 9.3%), the median (IQR) time to death was 1.7 (0.5–5.4) months overall (i.e., 50% of deaths by 12 months occurred within the first 1.7 months), with 63.0% (133/211) and 75.4% (159/211) of these deaths by 12 months occurring within the first 3 and 6 months, respectively. The median time to death was shortest and the proportion of the deaths that had occurred by 3 months was highest for children with SAM (0.7 month; *N* = 20/23 [87.0%]) followed by other conditions (1.3 months; *N* = 62/92 [67.4%]), severe anemia (2.1 months; *N* = 25/40 [62.5%]), severe pneumonia (2.6 months; *N* = 26/45 [57.8%]), and severe malaria (4.7 months; *N* = 0/11 [0·0%]) (Table 2).

### Overlapping conditions.

**Figure 2.**
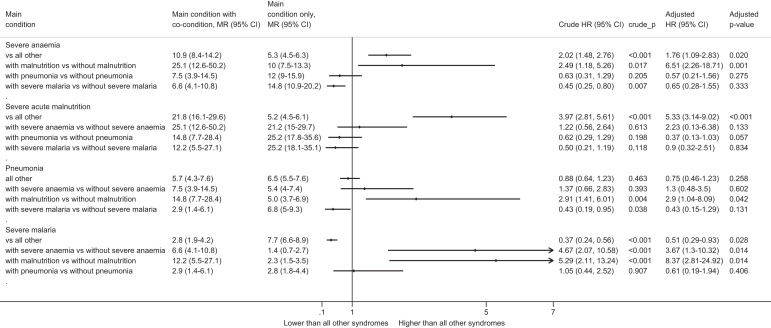
Six-month post-discharge mortality by main health condition relative to overlapping health conditions. There were no deaths for overlapping conditions between “other conditions” and severe anemia or severe malaria. Adjusted estimates were obtained from multivariable models that included the following covariates: age at admission, Hb levels on admission, bed net use, socioeconomic status, distance to hospital, maternal educational level, and mother’s HIV status. Hb = hemoglobin; HR = hazard ratio; MR = mortality rate; other conditions not included.

The co-presence of SAM was associated with significantly higher 6-month post-discharge mortality in children with severe anemia compared with severely anemic children who did not have SAM (MR = 25.1% versus MR = 10.0%, crude HR = 2.49, 1.18–5.26, *P* = 0.017) (Figure 2, Supplemental Figure 1, and Supplemental Table 1). The coexistence of SAM also increased 6-month post-discharge mortality in children with severe pneumonia (MR = 14.8% versus MR = 5.0%, crude HR = 2.91, 1.41–6.01, *P* = 0.004) or with severe malaria (MR = 12.2% versus 2.3%, crude HR = 5.29, 2.11–13.24, *P* < 0.001). The coexistence of severe anemia did not significantly increase 6-month mortality in SAM children (MR = 25.1% versus 21.2%, crude HR = 1.22, 0.56–2.64, *P* = 0.613) or children with severe pneumonia (MR = 7.5% versus 5.4%, crude HR = 1.37, 0.66–2.83, = 0.393), but it increased it in those with severe malaria (MR = 6.6% versus 1.4%, crude HR = 4.67, 2.07–10.58, *P* < 0.001) compared with children with other severe malarial conditions; that is, severe malarial anemia was associated with higher post-discharge mortality than severe malaria without severe anemia. By contrast, the coexistence of severe malaria was associated with lower 6-month post-discharge mortality in children with severe anemia (MR = 6.6% versus 14.8%, crude HR = 0.45, 0.25–0.80, *P* = 0.007); that is, children with severe malarial anemia had a lower risk of dying after discharge than children with severe non-malarial anemia. Severe pneumonia did not influence mortality in children with severe anemia, SAM, or severe malaria (Figure 2).

### In-hospital versus post-discharge mortality.

Overall, the odds of dying within the first 6 months after discharge were higher than during the in-hospital period for children admitted with severe anemia (MHOR = 2.20, 1.19–4.04, *P* = 0.011) and those admitted with other conditions (MHOR = 2.67, 1.71–4.15, *P* < 0.001). They were also higher for children with SAM (MHOR = 1.83, 0.91–3.70, *P* = 0.091) and severe pneumonia (MHOR = 1.81, 0.98–3.34, *P* = 0.056), but this difference was not statistically significant. There was no evidence of a difference in in-hospital and 6-month post-discharge mortality for children admitted with severe malaria (MHOR = 0.60, 0.14–2.51, *P* = 0.484) (Supplemental Figure 2).

### Other risk factors for post-discharge mortality.

In the univariate model, post-discharge mortality by 6 months changed with age (Table 3, Supplemental Figure 3) from MR = 9.5% in infants aged < 6 months to MR = 7.0% in the 6- to < 18-month-old group (HR = 0.72, 0.51–1.02, *P* = 0.065) and MR = 4.1% in the 36- to < 60-month-old group (HR = 0.36, 0.20–0.66, *P* = 0.001). Children exposed to HIV were twice as likely to die by 6 months compared with HIV-unexposed children (HR = 2.56, 1.61–4.07, *P* < 0.001). Post-discharge mortality decreased with increasing SES from MR = 10.1% in the poorest terciles to MR = 5.4% in the highest tercile (HR = 0.52, 0.36–0.77, *P* = 0.001). Higher maternal education was also associated with lower post-discharge mortality (secondary level education [MR = 5.3%] versus lower primary education [MR = 9.9%] [HR = 0.48, 0.24–0.95, *P* = 0.036]). Children in the tercile that lived farthest (third tercile) from the admitting hospital had a more than 2-fold higher post-discharge mortality compared with those in the first tercile (HR = 2.54, 1.70–3.80, *P* < 0.001). Bed net use was associated with lower 6-month post-discharge mortality (HR = 0.68, 0.51–0.91, *P* = 0.010). Treatment with antibiotics during in-patient care was not associated with post-discharge mortality (HR = 0.92, 0.67–1.26, *P* = 0.609). Among children with severe anemia, lack of receipt of blood transfusion was not associated with statistically significantly increased post-discharge mortality (HR = 1.64, 0.21–12.79, *P* = 0.636). However, a higher Hb level (≥ 4.0 g/dL) on admission (before receipt of any blood transfusion) was associated with a 55% lower risk of 6-month post-discharge mortality (HR = 0.45, 0.28–0.73, *P* = 0.001). Sex and discharge during malaria season were not associated with post-discharge mortality.

**Table 3:** Risk factors for 6-month post-discharge mortality

Exposure conditions	Univariate, *n/N* (%)	Univariate HR (95% CI)	*P*	Multivariate, *n/N* (%)	Multivariate HR (95% CI)	*P*
Age at admission (months)						
< 6	51/180 (28.3)	1.00	–	51/180 (28.3)	1.00	–
6 to < 18	84/180 (46.7)	0.72 (0.51–1.02)	0.065	84/180 (46.7)	0.62 (0.35–1.11)	0.108
18 to < 36	32/180 (17.8)	0.41 (0.27–0.64)	< 0.001	32/180 (17.8)	0.37 (0.19–0.73)	0.004
36 to < 61	13/180 (7.2)	0.36 (0.20–0.66)	0.001	13/180 (7.2)	0.43 (0.17–1.08)	0.074
Admission Hb (< 4.0 g/dL)	161/180 (89.4)	1.00	–	161/180 (89.4)	–	–
Admission Hb (≥ 4.0 g/dL)	19/180 (10.6)	0.45 (0.28–0.73)	0.001	19/180 (10.6)	1.41 (0.54–3.71)	0.487
Slept under bed net (no)	89/180 (49.4)	1.00	–	89/180 (49.4)	1.00	–
Slept under bed net (yes)	91/180 (50.6)	0.68 (0.51–0.91)	0.010	91/180 (50.6)	0.73 (0.45–1.18)	0.201
Sex (male)	80/180 (44.4)	1.00	–	–	–	–
Sex (female)	100/180 (55.6)	1.11 (0.83–1.50)	0.468	–	–	–
Discharge season (no malaria)	95/180 (52.8)	1.00	–	–	–	–
Discharge season (malaria)	85/180 (47.2)	0.96 (0.72–1.29)	0.785	–	–	–
Blood transfusion (no)	34/84 (40.5)	1.00	–	–	–	–
Blood transfusion (yes)	50/84 (59.5)	1.64 (0.21–12.79)	0.636	–	–	–
Antibiotics prescribed (no)	58/180 (32.2)	1.00	–	–	–	–
Antibiotics prescribed (yes)	122/180 (67.8)	0.92 (0.67–1.26)	0.609	–	–	–
Socioeconomic status						
First tercile–poorest	77/176 (43.8)	1.00	–	77/176 (43.8)	1.00	–
Second tercile	58/176 (33)	0.76 (0.54–1.07)	0.112	58/176 (33)	1.20 (0.72–2.01)	0.480
Third tercile	41/176 (23.3)	0.52 (0.36–0.77)	0.001	41/176 (23.3)	0.69 (0.39–1.25)	0.221
Distance from hospital						
First tercile–nearest	33/180 (18.3)	1.00	–	33/180 (18.3)	1.00	–
Second tercile	64/180 (35.6)	1.97 (1.30–3.00)	0.002	64/180 (35.6)	1.60 (0.84–3.03)	0.150
Third tercile	83/180 (46.1)	2.54 (1.70–3.80)	< 0.001	83/180 (46.1)	1.98 (1.05–3.75)	0.040
Mother’s educational level						
Lower primary	10/178 (5.6)	1.00		10/178 (5.6)	1.00	–
Upper primary	110/178 (61.8)	0.66 (0.34–1.25)	0.201	110/178 (61.8)	0.47 (0.21–1.06)	0.069
Secondary	50/178 (28.1)	0.48 (0.24–0.95)	0.036	50/178 (28.1)	0.40 (0.17–0.99)	0.046
Tertiary	8/178 (4.5)	0.62 (0.24–1.59)	0.320	8/178 (4.5)	0.64 (0.14–2.89)	0.565
Mother’s HIV status (negative)	25/86 (29.1)	1.00	–	25/86 (29.1)	1.00	–
Mother’s HIV status (positive)	61/86 (70.9)	2.56 (1.61–4.07)	< 0.001	61/86 (70.9)	2.12 (1.26–3.57)	0.005

Hb = hemoglobin; HR = hazard ratio. The multivariable model adjusts for all the other variables mentioned in the table except for discharge season, blood transfusion, and antibiotics.

## DISCUSSION

In this high–malaria transmission and disease burden area in rural western Kenya, children admitted with severe anemia and SAM were 2.6 and 1.8 times more likely to die in-hospital and 2.6 and 4 times more likely to die within the first 6 months after discharge than children admitted for other reasons. Overall, about one in five children (20.7%) with severe anemia and one in four children (24.7%) with SAM died in the year after discharge, which was much higher than the 5.9% and 9.2% during the in-hospital period; 62% and 87% of these post-discharge deaths occurred in the first 3 months, stressing the importance of the urgent need for post-discharge strategies to address this risk period.

Our findings are consistent with previous observational studies in similar study areas in Kenya,^7^ Malawi,^6^ and Uganda,^5^ which reported comparable high rates of post-discharge mortality among children admitted with all-cause severe anemia. Of note is that these rates are considerably higher than those previously reported in the placebo arms of three PDMC trials in children with severe anemia in Malawi^14^^,^^15^ and Kenya.^16^ It is likely that the children in these trials benefitted from access to better in-hospital care and closer follow-up than in routine settings. Overall, factors associated with post-discharge mortality were also comparable with earlier reports from similar epidemiological settings, including young age, HIV exposure, low SES or maternal education, distance from the hospital, and Hb levels on admission.^6^^,^^17^ Unfortunately, HIV status was not available for 94.5% of these children.

The time to post-discharge death differed between conditions (Table 2). In children with SAM, 87% of total deaths by 12 months occurred within the first 3 months after discharge. This was approximately 62% for children with severe anemia and 57% for children with severe pneumonia. By contrast, for children with severe malaria, the time to death was more spread over the year with a median survival time of 4.7 months. The first few months after discharge have been recognized as a period of high vulnerability to mortality and morbidity in children with SAM and severe anemia, possibly because of physiological stress and impairment of the immune system.^17^^–^^20^ In malaria-endemic areas, *Plasmodium falciparum* infection has been shown to lead to endothelial inflammation, which may persist for more than a month even after the parasites have been cleared.^21^ This is likely to predispose to or worsen untreated bacterial infections,^22^ including invasive non-typhoidal *Salmonella* (iNTS) infections, which have been shown to have a positive correlation with *P. falciparum* infection in malaria-endemic areas in sub-Saharan Africa.^23^ Children with coexisting iNTS and falciparum malaria infections have a high in-hospital MR^24^ that is even worse in children with severe malarial anemia^25^ and likely contributes to high mortality in the immediate post-discharge period.

The high risk of post-discharge mortality in children with SAM and severe anemia was also evident in our analysis of the impact of the coexistence of multiple conditions. Overall, we found that children with severe pneumonia and severe malaria had the worst outcomes if they also had SAM or severe anemia. For example, 6-month post-discharge mortality was about 5-fold higher in children with severe malaria if they also had SAM or severe anemia. Similarly, children with severe pneumonia had a 3-fold higher mortality if they also had SAM.

Owing to insufficient data, we could not explore the causes of post-discharge death. These are likely multifactorial in this setting, including HIV infection, other acute and chronic infections (e.g., tuberculosis), and malnutrition or micronutrient deficiencies. However, previous studies in similar epidemiological settings among children admitted with severe anemia have reported malaria as the main reason for morbidity and mortality in the post-discharge period. In Uganda,^5^ 89% of rehospitalizations and 59% of clinic visits were due to malaria, and in previous studies in this same study area in western Kenya, 30% of post-discharge deaths were due to malaria.^7^ In a similar setting in Kenya, 50% of participants were parasitemic 1 month after discharge.^8^ Full recovery from malaria-associated anemia can take up to 6 weeks, possibly because of continued dyserythropoiesis and bone marrow suppression and new infections or recrudescence due to poor parasite clearance.^26^ Further studies are needed to elucidate the non-malarial causes of severe anemia and post-discharge readmissions and death.

Another noticeable finding was the relatively low risk of post-discharge mortality in children with severe malaria without SAM or severe anemia. Severe malaria is an acute condition in which proper diagnosis and treatment result in a better prognosis than if children are admitted with other causes of severe anemia in this setting. The etiology of non-malarial severe anemia is complex, multifactorial, and more difficult to diagnose and thus treat appropriately because of limited human and structural capacity in the healthcare system.^27^ In many lower-level health facilities in malaria-endemic areas, malaria and anemia are diagnosed clinically following the Integrated Management of Childhood Illnesses guidelines.^28^ Parenteral antimalaria treatment and blood transfusion may be administered presumptively, mostly without further investigation for other possible underlying conditions. Therefore, inadequate care during the in-hospital stay due to challenges in diagnosis and treatment may result in disease progression and poor post-discharge prognosis among children with non-malarial severe anemia. Another possible reason reported in other studies is the misclassification of uncomplicated malaria as severe malaria, therefore underestimating the severe malaria–specific MRs; it has been shown that about a third of admissions for “severe malaria” do not fulfill WHO’s criteria for severe malaria in malaria-endemic sub-Saharan Africa.^29^

Almost all post-discharge deaths in this study (> 95%) occurred in the community before children were referred or could reach the hospital. These findings are consistent with a recent study in an HDSS area in Mozambique, where > 80% of post-discharge deaths in children were reported to have occurred outside the hospital,^17^ and in another study among children admitted with infectious conditions in Uganda, where 67% of post-discharge deaths occurred in the community.^30^^–^^32^ We found lower maternal education to be associated with increased post-discharge mortality. Thus, an intervention that includes maternal/caretaker education at discharge on how to identify the danger signs and seek prompt care may help reduce the burden. This is further supported by the above-mentioned finding that post-discharge MRs in the placebo arm of the PDMC trial conducted in this area^16^ were much lower than in this observational study, similar to the differences in MRs between trials and observational cohort studies in Malawi.^6^^,^^14^

A major limitation of this study was the lack of data on the HIV status of participants. At the time of the study, testing for HIV among the pediatric population in Kenyan public hospitals was not offered routinely. Although information on maternal HIV status was available for many (71.4%), data on the actual HIV status of the child were available in only 5.5%. The study area is known to have a high prevalence of HIV infection, and pediatric HIV infection is a major risk factor for severe anemia.^33^ Studies in areas with high HIV and malaria co-infection rates in Uganda and Kenya have reported up to 5-fold higher 6-month post-discharge MRs in children with severe malarial anemia associated with HIV infection.^34^ Another major limitation is the lack of additional information about other comorbidities associated with the primary diagnosis of severe anemia. The etiology of severe anemia in this setting is complex and multifactorial, including nutritional deficiencies; hookworm infestations; acute and chronic infections such as malaria, bacteremia, tuberculosis, and HIV infection; and genetic disorders such as sickle cell anemia.^1^ There was also limited information on diarrhea and sepsis, which cause substantial morbidity in sub-Saharan Africa.^35^ The latter reflects the limited diagnostic capacity in Kenyan public hospitals. Many children with sepsis may have ended up in the other conditions group in our analysis. Furthermore, the diagnoses were based on the prevailing national guidelines from the Kenya Ministry of Health, which may differ slightly from international definitions from the WHO. This may have resulted in the potential misclassification of some children and may affect the generalizability of the findings. Lastly, childhood vaccination status, a potential confounder of the risk of post-discharge mortality, was not available.

The results of this study add to the limited evidence from other studies in malaria-endemic areas and confirm that children under 5 years of age admitted with SAM and severe anemia are at a high risk of post-discharge mortality in the first few months after discharge. Guidelines exist for the management of children with SAM after discharge. Similar guidelines for the post-discharge management and follow-up of children with severe anemia were recently published by the WHO for malaria-endemic areas and now need to be implemented. Furthermore, studies of predictors and the etiology of post-discharge mortality are urgently needed to develop targeted management guidelines for post-discharge care.

## Supplemental Materials


Supplemental materials
