# Supplementary material for: Post-Discharge Risk of Mortality in Children under 5 Years of Age in Western Kenya: A Retrospective Cohort Study
Source: Am J Trop Med Hyg. 2023 Aug 7;109(3):704–12. doi: 10.4269/ajtmh.23-0186 (PMC10484264; doi:10.4269/ajtmh.23-0186)
Supplement: Supplementary file 1 [file tpmd230186.SD1.pdf]

Supplementary Appendix

This appendix has been provided by the authors to give readers additional information about their work.

Supplement to:

Titus K Kwambai, Meghna Desai, Menno Smit, Sarah Nevitt, Eric Onyango, Simon Kariuki, Martina Oneko, Aaron Samuels, Mary Hamel, Feiko O ter Kuile.

Supplemental tables

Table S1: Mortality at 3, 6- and 12-months post-discharge by condition combination

Condition	3 months post-discharge mortality	6 months post-discharge mortality	12 months post-discharge mortality
SA	8.0% (47/585)	11.3% (61/541)	16.1% (78/483)
SM	1.8% (17/953)	3.0% (27/890)	5.7% (45/787)
PN	4.1% (38/937)	5.9% (52/882)	8.9% (71/801)
SAM	15.0% (35/234)	19.5% (42/215)	22.1% (43/195)
OT	4.6% (65/1421)	5.8% (77/1338)	8.2% (98/1189)
SA_SM	5.2% (14/268)	7.3% (18/246)	12.3% (27/219)
SA_PN	4.4% (6/137)	6.9% (9/131)	8.4% (10/119)
SA_SAM	17.5% (7/40)	24.2% (8/33)	28.6% (8/28)
SA_OT	0.0% (0/10)	0.0% (0/10)	0.0% (0/9)
SM_PN	1.1% (3/264)	2.8% (7/249)	3.4% (8/236)
SM_SAM	7.1% (4/56)	12.2% (6/49)	14.0% (6/43)
SM_OT	0.0% (0/17)	0.0% (0/16)	0.0% (0/14)
PN_SAM	7.1% (5/70)	14.9% (10/67)	16.1% (10/62)
PN_OT	0.0% (1/19)	2.0% (3/19)	2.0% (4/17)
SAM_OT	33.3% (2/6)	33.3% (2/6)	40.0% (2/5)
SM_PN_SAM	0.0% (0/22)	10.0% (2/20)	11.1% (2/18)
SM_PN_OT	0.0% (0/2)	0.0% (0/2)	0.0% (0/2)
SA_PN_SAM	7.7% (1/13)	18.2% (2/11)	25.0% (2/8)
SM_SAM_OT	0.0% (0/1)	0.0% (0/1)	0.0% (0/1)
SA_PN_OT	0.0% (0/1)	0.0% (/1)	0.0% (/1)
SA_SM_SAM	11.1% (2/18)	20.0% (3/15)	25.0% (3/12)
PN_SAM_OT	0.0% (0/1)	0.0% (0/1)	0.0% (0/1)
SA_SAM_OT	0.0% (0/0)	0.0% (0/0)	0.0% (0/0)
SA_SM_OT	0.0% (0/2)	0.0% (0/2)	0.0% (0/2)
SA_SM_PN	0.0% (2/73)	0.0% (0/69)	0.0% (0/66)
SA_SM_SAM_OT	0.0% (2/0)	0.0% (3/0)	0.0% (3/0)
SA_PN_SAM_OT	0.0% (0/0)	0.0% (0/0)	0.0% (0/0)
SA_SM_PN_OT	0.0% (0/0)	0.0% (0/0)	0.0% (0/0)
SA_SM_PN_SAM	0.0% (0/7)	16.7% (1/6)	25.0% (1/4)
SM_PN_SAM_OT	0.0% (0/0)	0.0% (0/0)	0.0% (0/0)
SA_SM_PN_SAM_OT	0.0% (0/0)	0.0% (0/0)	0.0% (0/0)

SA, severe anaemia; SM, severe malaria; PN, severe pneumonia; SAM, severe acute malnutrition; OT, `Other'. OT denote the undefined conditions excluding SA, SM, PN or SAM.

Supplemental figures

Figure S1: Overlapping conditions and 6-month post-discharge mortality

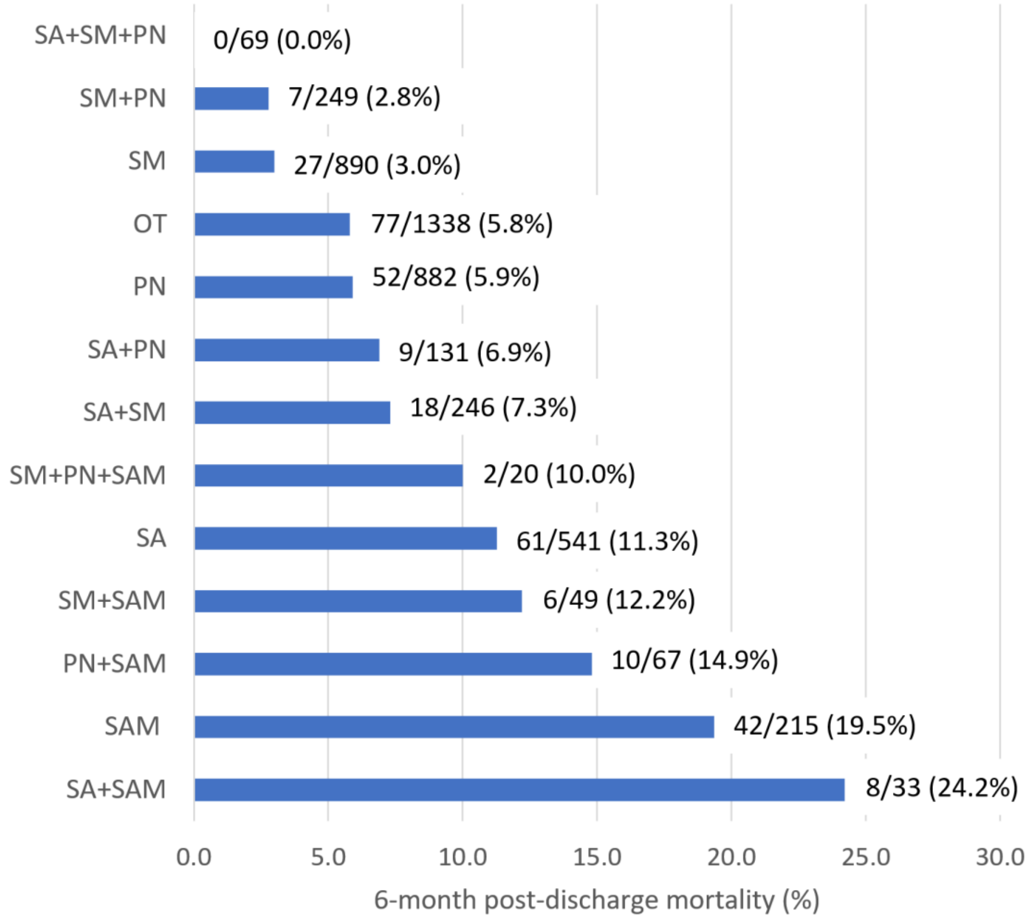

SA, severe anaemia; SM, severe malaria; PN, severe pneumonia; SAM, severe acute malnutrition; OT, `Other'. OT denote the undefined conditions excluding SA, SM, PN or SAM.
6-month post-discharge mortality for conditions with 20 or more patients per subgroup

Figure S2: In-hospital vs post-discharge mortality by condition by six months

Admission condition	In-hospital deaths n/N (%)	Post-Discharge deaths n/N (%)	MHOR (95% CI)	p
Severe anaemia	15/255 (5.9%)	33/213 (15.5%)	2.20 (1.19, 4.05)	0.011
Severe acute malnutrition	12/130 (9.2%)	22/102 (21.6%)	1.83 (0.91, 3.70)	0.091
Severe malaria	5/499 (1.0%)	3/433 (0.7%)	0.60 (0.14, 2.51)	0.484
Severe pneumonia	16/586 (2.7%)	29/514 (5.6%)	1.81 (0.98, 3.34)	0.056
Other syndromes	27/1468 (1.8%)	72/1294 (5.6%)	2.67 (1.71, 4.15)	<0.001

NOTE: Weights are from random effects analysis

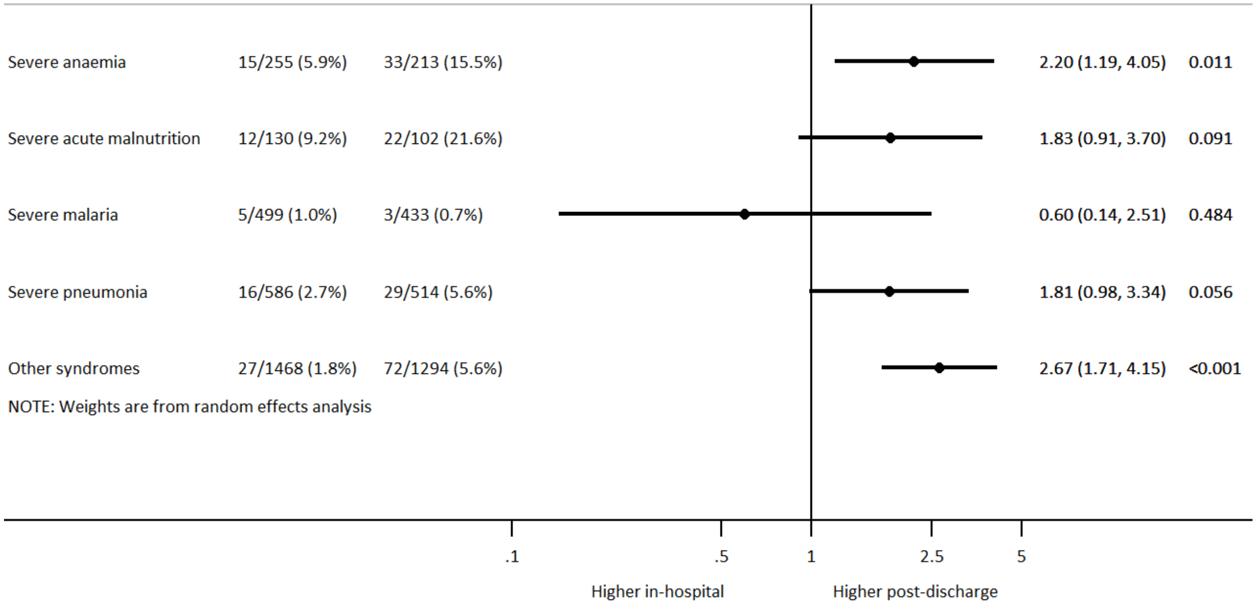

MHOR, Mantel-Haenszel odds ratio; CI, confidence interval; `Other conditions` denote the undefined conditions in children that did not have severe anaemia, severe malaria, severe pneumonia, or severe acute malnutrition (SAM).

Figure S3: Kaplan Meier curve showing post-discharge mortality by age category

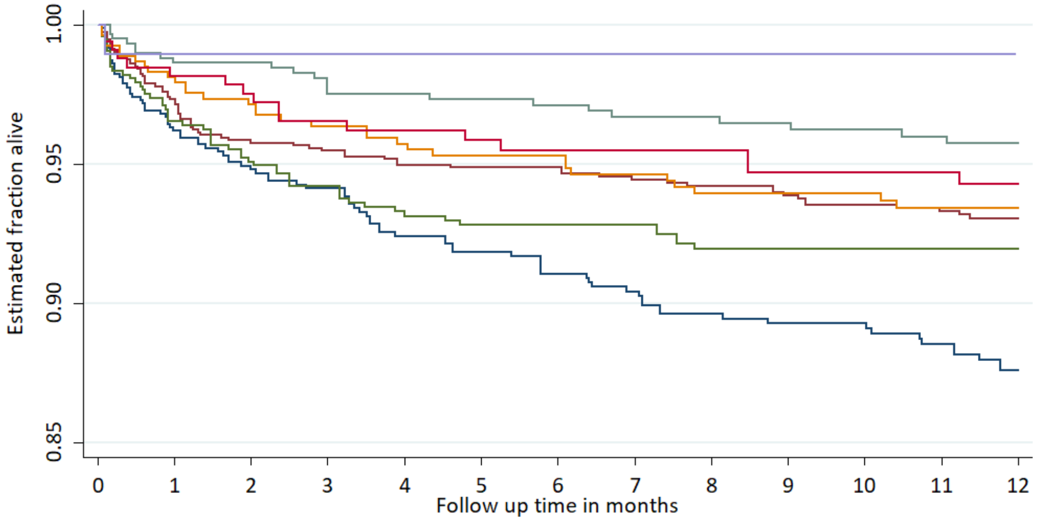

Number at risk	0	2	4	6	8	10	12
<6 months	846	723	641	583	528	493	447
6 to <12 months	1132	1011	943	900	845	797	748
12 to <18 months	723	650	607	556	526	504	482
18 to <24 months	535	492	460	425	395	377	361
24 to <36 months	590	547	508	456	426	407	394
36 to <48 months	324	304	280	259	241	228	217
48 to 60 months	189	157	128	89	61	40	0

Number at risk at time ‘0’ denotes the number of children per age category who were discharged alive from hospital.
